# A qualitative investigation of mental health in women of refugee background resettled in Tasmania, Australia

**DOI:** 10.1186/s12889-021-11934-y

**Published:** 2021-10-18

**Authors:** Clare Hawkes, Kimberley Norris, Janine Joyce, Douglas Paton

**Affiliations:** 1 College of Health and Human Sciences, Charles Darwin University, Ellengowan Drive, Casuarina, Darwin, NT 0811 Australia; 2 School of Medicine (Psychology), University of Tasmania, Hobart, TAS 7001 Australia

**Keywords:** Women, Refugee, Resettlement, Mental health, Australia, Regional

## Abstract

**Background:**

Women of Refugee Background (WoRB) are a highly vulnerable population with complex going mental health needs following resettlement. In Australia, there has been a substantial increase in WoRB being resettled in rural and regional locations. Despite this, no research to date has specifically focused on factors contributing to mental distress in WoRB in regional resettlement locations. The current study aimed to address this gap in literature.

**Methods:**

21 semi-structured qualitative interviews were conducted with WoRB and service providers in regional locations of Tasmania, Australia. Interviews were audio recorded and transcribed verbatim. Transcripts were analysed utilising Braun and Clarke (Qual Res Psychol 3(2):77–101, 2006) framework for conducting thematic analysis.

**Results:**

Thematic analysis revealed that WoRB conceptualised mental health as a pathogenic entity, which significantly influenced their mental health help-seeking behaviours. The findings also highlighted how resettlement to a rural and regional location of Australia may exacerbate many of the factors which contribute to ongoing mental distress in WoRB.

**Conclusions:**

The findings of the current study build upon existing research which indicates the adverse impacts post-migrations stressors can have on the mental health of individuals of refugee background. Furthermore, this study suggests that the current services and supports available to WoRB resettled in regional locations of Australia are inadequate, and under-resources. These findings are discussed in regard to practical and policy implications which should be addressed to better support the mental health of WoRB resettled in rural and regional locations of Australia.

Women of Refugee Background (WoRB) are a particularly vulnerable and understudied refugee population [1]. Throughout all stages of migration, WoRB are at significant risk of experiencing gender-based vulnerabilities and threats including sexual assault, and complex post-migration problems and socio-cultural disadvantages such as limited social support and greater language barriers [2, 3]. The prolonged and dynamic nature of these experiences, and their interaction, creates significant long-term mental health implications [1]. Research indicates that WoRB experience significant levels of psychopathology, including greater levels of PTSD [4–6], anxiety [7], and depression [5]. WoRB have also been identified to experience higher levels of psychological distress than males of refugee background during the initial years of resettlement [8], with this high degree of distress persisting throughout the initial years of resettlement [4]. Despite acknowledgment of this greater risk, WoRB are profoundly under-represented amongst those seeking mental health supports in resettlement countries [9]. Furthermore, front line Western based interventions, such as psychotherapy, have limited utility in reducing distress in this population [10]. The combination of high levels of psychopathology and low levels of help seeking and standard treatment efficacy makes it imperative that the nature and the predictors of mental health in WoRB are understood in resettlement countries, including Australia [11]. Gaining a deeper understanding of the mental health needs of WoRB will allow policy makers and service providers to respond effectively to this vulnerable group [12], including developing training in population-specific treatments for health and community professionals. The development of this capability is an important adjunct to resettlement planning in countries, such as Australia, that specifically support WoRB resettlement.

## Women of refugee background in Australia

Consistent with the United Nations High Commissioner for Refugees (UNHCR) resettlement policies [13], Australia priorities the resettlement of WoRB, and can be identified as one of the few countries in the world which allocates a specific resettlement quota for women identified as being at risk and in need of urgent protection [14]. Australia adopted the UNHRC “Women at Risk” program at its inception in 1989. Since this time, the Australian Government has continued to resettle WoRB identified as particularly vulnerable, with a total of 2345 visas being granted via this visa pathway in the 2019–2020 humanitarian program [15]. Coinciding with the overall increase in resettlement of WoRB in Australia, the number of WoRB resettling in rural and regional locations of Australia has also increased substantially [16].

## Women of refugee background and regional and rural resettlement

As a whole, research investigating the mental health of individuals of refugee background resettled in rural and regional locations in Australia has been reported as being practically non-existent [17, 18]. The small body of research which has focused on the topic has suggested that, compared to their male counterparts, WoRB resettled in regional locations are not only at higher risk of experiencing mental health difficulties such as depression (14.3% vs, 41.2%, respectively) [19], but also at high risk of experiencing predisposing and perpetuating factors which increase the likelihood of experiencing negative mental health outcomes. This includes lower levels of education and literacy, which impacted their capacity to engage in education and develop their language skills [18]. WoRB also expressed high levels of isolation, loneliness, and lack of social networks [20] and were more likely to experience additional isolation and loneliness due to staying at home in a primary caregiver role looking after children due to a lack of financial resources to afford childcare. This had further flow-on effects, resulting in greater isolation and a lower likelihood of developing language skills [20, 21]. Despite these studies suggesting that WoRB may be at particular risk of experiencing higher levels of psychopathology and ongoing mental health challenges during resettlement in rural and regional locations, no study to date can be identified as specifically focusing on the mental health of WoRB resettled in a rural or regional location of Australia [22]. This is a notable deficit in research and practice, particularly in a context of ever-increasing numbers of WoRB being resettled in Australia and a growing number of WoRB on the Women at Risk Visa being resettled in regional locations [16]. This increase in resettlement of WoRB to rural and regional areas also increases the number of WoRB who are likely to come into contact with mental health services in rural and regional locations. To effectively cater for this population and the anticipated growth in WoRB numbers, practitioners must increase their understanding of the diverse characteristics of WoRB refugees and the contextual factors which impact their mental health when being settled in the rural and regional location of Australia. To date, this research does not exist. Therefore, the current study aimed to address this shortcoming in the literature by exploring conceptualisations of mental health, and identify key factors influencing mental health, in WoRB resettled in a regional location of Australia.

## Method

### Design

A qualitative methodological framework was utilised in the current research, consisting of individual semi-structured interviews guided by a set of open-ended questions (Table 1). This framework was selected so rich and in-depth information could be collected, in order to gain a detailed description of how mental health is conceptualised in WoRB, and key factors contributing to the mental health of WoRB resettled in a regional location. All participants had the opportunity to review the questions prior to the interview beginning and opt out of answering any questions that did not feel comfortable answering. No participants opted out of any initial questions.

**Table 1:** Initial Open-Ended Guiding Questions

- How do you understand mental health in Women of Refugee Background in Tasmania? (Service Provider/Volunteer)	
- What does mental health mean to you? (Women of Refugee Background)	
- What factors contribute to mental health in Women of Refugee Background?^a^	
- What are the mental health needs of Women of Refugee Background in Tasmania?^a^	
- How do services address the mental health needs of Women of Refugee Background in Tasmania?^a^	
- What factors impact the ability for services to address the mental health needs in Women of Refugee Background?^a^	

^a^asked to all participants (Women of Refugee Background or Service Provider/Volunteer

### Setting

The current study was conducted in Tasmania, Australia. Tasmania is the island south of mainland Australia, and has a population of 509,965 [23], with the entire island holding rurality classifications ranging between regional and very remote. Tasmania is also identified as the least multi-cultural population in Australia, with 80.7% of its residents being born in Australia, from European ancestry [24]. Tasmania takes a higher proportion of Humanitarian entrants relative to its overall migrant intake, with 25–32% of migrants arriving in Tasmania entering on humanitarian visas with over one quarter of these entrants being on the ‘Women at risk’ 204 visa [16]. Tasmania is also the resettlement location of over 10% of Australia’s intake of Women at Risk [16]. As such, Tasmania provides a useful context in which to examine the impacts of resettlement in rural and regional locations which differ substantially from more metropolitan regions typical of other Australia states.

### Ethical consideration

Research involving individuals of refugee backgrounds poses particular ethical challenges [25]. One of the core challenges is voluntary, informed consent. This is impacted by limited English fluency and comprehension, varying levels of literacy in participants own language and suspiciousness of written consent forms due to different cultural traditions [25, 26]. Due to limited English fluency and comprehension, research involving individuals from a refugee background may use interpreters. Although this increases the number of potential participants, due to reducing the need for the participant to have a certain degree of English comprehension and fluency, it does reduce the researcher’s ability to uphold confidentiality, as other individuals (i.e., interpreters) have been involved in the data collection process. This can be addressed in research by having the interpreters sign a confidentiality agreement [25], The current study addresses any concerns surrounding confidentiality and using interpreters by conducting the interview in English. Additional factors influencing this decision was the small population of individuals of refugee background resettled in Tasmania. It was identified in interviews involving service providers that using interpreters can be a barrier to seeking mental health support, as some individual express concerns surrounding the confidentiality. This is particularly important when discussing topics surrounding mental health, due to stigma. Tasmania can also be identified as having a very small number of WoRB, in comparison to major metropolitan locations. This increases the likelihood of a potential participant would know the interpreter utilised in the research process, and thus bringing into question the confidentiality of the data collected. These ethical challenges were addressed by the selection criterion of being able to speak English at a level where the interview could be conducted in English. This was implemented to increase the likelihood of the WoRB understanding the purposes of the study, the associated risks and benefits, and thus allowing them to provide informed consent. All participants were provided with a plain language statement in English outlining the purposes of the study, with the researcher conducting the interviews spending time verbally explaining the information pertaining to the study and answering any questions the participants had prior to gaining consent. To address the potential barrier of written consent forms, WoRB were provided with the option to give either written consent, oral consent, or both, with 7 out of the 9 WoRB opting to provide oral consent to participate in the study. Ethics approval for the current study was obtained through the Tasmanian Social Sciences Human Research Ethics Network (H0017941; H0020021) and Human Research Ethics Committee at Charles Darwin University (H19003; H19087).

### Sampling, recruitment and participants

Recruitment for the current study employed purposive sampling techniques in the initial stages, with participants being consciously selected on the basis of their capacity to contribute to the research and comprised of both individuals who identified as WoRB, as well as volunteers and service providers who support WoRB. Service providers and volunteers were invited to participate in the current study as they play a key role in supporting WoRB during the early stages of resettlement, and influence factors associated psychological well-being, including meeting daily living needs and gaining awareness of, and access to, support services [27], thus they are able to provide vital insight into factor that they observe as being predictors of mental health in WoRB.

Participants were initially identified via contacting refugee support agencies in Tasmania listed on the Refugee Council of Australia website via email. This was a generic email sent from the university email account of the first author, requesting expressions of interest to participate in the study. The email clearly outlined that the research was independent of the organisation, and participation was voluntary and anonymous. Individuals interested in participating contacted the first author by phone, or email. Subsequent participants were identified via snowball sampling techniques. All participants needed to be over the age of 18 and speak a level of conversational English which allowed the interview to be completed in English. The location and time of the interview was determined by a conversation occurring via the telephone. Interviews were conducted in services, participant homes and private study rooms at public libraries. WoRB who agreed to participate in the study were offered a $20 gift voucher as compensation for their time. Participants were informed that they were able to withdraw from the study at any time, with no impact to them receiving the gift voucher. All interviews were conducted between May 2019 and August 2020.

A total of 21 individuals participated in the interviews (nine WoRB, four individuals in volunteer-based roles and eight service providers [in paid roles] who support WoRB during resettlement). Further participant demographic information was not collected, and hence not reported, to ensure confidentiality and anonymity of participants, due to the regional location, and the participants having unique characteristics (i.e., being a WoRB or working/volunteering for one of the limited refugee support services), thus making them more easily identifiable to local stakeholders, than members of the general population.

All interviews were conducted by the first author (a female clinical psychologist and PhD student), ranging from 45 to 75 min in length, and were audio recorded. Audio-recordings from interviews were transcribed verbatim and interviewees were provided the opportunity to review the transcript for comment and/or correction.

### Data analysis

Data saturation, the point where no new interview themes emerged, determined the final number of interviews required for the study. To identify when saturation occurred, data was continuously analysed throughout the data collection period utilsing Nivo 12 software. An audit trail was kept throughout the research process to aid the researcher in identifying when data saturation was reached. Within the current study, by interview 17, the audit trail entries illustrated that the list of new themes began to decline, until there were no new themes identified from the 21st interview, hence, this was deemed the last interview.

As the current research focused on reporting the experiences and reality of participants, transcripts were analysed utilising Braun and Clarke [28] six-step framework for conducting thematic analysis, utilising inductive thematic description at the semantic level which was further underpinned by an essential/realist approach. NVivo qualitative data analysis software (version 12) was utilised for data management. Reporting was guided by the Consolidated Criteria for Reporting Qualitative Research (COREQ) [29].

## Results

The results are discussed aligning with the two overarching themes identified within the data: Pathogenic Conceptualisation of Mental Health and Contributing Factors to Mental Distress in WoRB Resettled in a Regional location. The latter consisted of five sub-themes.

### Pathogenic conceptualisation of mental health

WoRB described the term ‘mental health’ as being associated with a form of pathology, or negative entity, which has a particular negative impact on the mind.

‘Mental health, the way I know how to explain is crazy, or stress, or mental is… talking alone, is too much stress. When you don’t have friends, you end up thinking too much’ (WoRB 2)

‘Something in your mind… it’s concerning’…(WoRB 4)

‘Mental health is, how do I say it, it’s hard to explain, mental health is depression and it is like, you don’t want to be depressed but it comes all the time’ (WoRB 3)

Service providers also highlighted and emphasised that the WoRB they support conceptualise mental health as a pathogenic entity/experience, which in turn had a substantial negative impact on WoRB accessing mental health services due to the stigmatising perceptions associated with their understanding of mental health as a construct.

‘Mental health is something that a lot of our women have a different understanding of – they come to Australia with their understanding of what mental health is, because sometimes what we hear from the women is “I’m not crazy! I don’t need to go to a psychologist. I don’t need to go to a counsellor.” And it’s like “it’s not about being crazy, you know, we all need support” (Service Provider 4)

‘Mental health, it’s a really stigmatised issue, and we’re talking about, like, the normative majority of this country,... and if they’re a migrant, then that’s even harder’. (Service Provider 7)

Likewise, WoRB expressed concerns that they would be seen as ‘crazy’ by other members of society if they sought assistance for their mental health concerns, which had negative, stigmatising ramifications.

‘You are crazy, they will keep in you a crazy place, even if you would be okay with some medicines, but they are worried that you will go crazy more…’ (WoRB 2)

‘Sometimes our people get really afraid of getting stigmatised, so they tend not to talk about their issues freely with other people. So that is a big issue that our people have been experiencing, even when they are aware of the support services in the society that they can access, they tend not to share their stories because of that fear as well’.

(WoRB 6)

WoRB and service providers identified barriers which contributed to mental health, and subsequently accessing mental health services, being perceived as stigmatising - in particular, how the community of origin may see you.

‘If you go to a service for help, you’re kind of – you know, there’s a lot of shame that comes along with that, you’re treated very differently within your community and they don’t like to speak about it’. (Service Provider 3).

‘You have these counselling things where you sit down with someone and you tell them all your business, and for a lot of people, they’re like, we don’t do that, you know, we don’t tell people our-our business. That’s just, you know, it’s embarrassing for the family; it’s shameful for the family to do that.’ (WoRB 8)

WoRB also highlighted how religion and faith can increase the stigma associated with accessing mental health support.

‘In my culture mental health isn’t, um, given a lot of importance, and people tend to, kind of, dismiss issues of mental health with, things like, you know, they’d say, if you prayed enough, you wouldn’t be sad, or that kind of thing, so there’s a lot of, like, relation between mental health and religion, and spirituality’ (WoRB 1).

‘So, if you are seen as having mental health problems, then you’re lacking in some way, you’re not religious enough, you’re not spiritual enough, you don’t have enough faith. And so, like, it is to admit that is a problem’ (WoRB 4)

Fear and stigma associated with accessing mental health services for support was a particular barrier identified in the current study focusing on WoRB resettled in a regional location, due to decreased anonymity, and very few services which they could access.

‘There are certain things that stop us. I think the [name removed] is much better … but people are scared to go to the centre. My people, they don’t like … there stuff getting splashed…and they think that if they go to the centre, the centre will splash it. people are just so worried about their family splashing … that’s it. For example, in my community, that is why people are not going to the centre’ (WoRB 5)

‘If you go to a service for help, your kind of – you know, there’s a lot of shame that comes along with that, you’re treated very differently within your community and they don’t like to speak about it’ (WoRB 3)

‘That’s a particular nuance in Tasmania that we struggle with. Some clients don't want to access our service because they know family members access other parts of our service and the anonymity and the need for that is key’ (Service Provider 1)

### Contributing factors to mental distress in WoRB resettled in a regional locations

The sub-themes that emerged under the broad theme of Contributing Factors to Mental Distress in WoRB Resettled in a Regional locations comprised of ‘Loneliness and Isolation’, ‘Trauma stemming from the Resettlement Process’, ‘Inability to Access Fundamental Needs for Everyday Life’, ‘Family Separation and Ongoing Concerns for Family and Friends in their Country of Origin’ and ‘Chronic Uncertainty’.

### Loneliness and isolation

WoRB emphasised the significant impact that isolation and loneliness have on mental health during resettlement.

‘And I feel lonely … like for 1 months let’s say… and it was giving me like stress … because morning I woke up… first eat and then sit…. And I was like, what happened? … So you feel lonely … and first of all I am lucky, I have two sons at least… but for some people who are coming alone, and they are use to having people, they end up having stress like they are crazy’ (WoRB 2)

‘Even if your fridge is full… it can’t make you happy because you a lonely…..’(WoRB 9)

‘The most stressful thing was that I become alone… for many many days …I was alone, just me and my sons… no one, they didn’t say hello to us… which means we just went to town and the house, town and house... and Saturday, Sunday, no church… no one can come and take us…it was full stress’ (WoRB 5)

‘If you live in a country and you don’t have a family member with you, or if you don’t have someone who can communicate with you, or have a chat with you, to go through your life with you, and this sort of stuff, or be around to give you advice. Like, if your lonely, there is such a high chance that you will be depressed. (WoRB 3)

Factors contributing to high levels of isolation and loneliness were identified as multifaceted, including gender based cultural norms from WoRB culture of origin, which were emphasised as being patriarchal in nature.

‘Ahh I think the isolation, and also the issue within the family, is because our people have a male dominant culture like you know, the male has control… and the male is the head of the house. I think that often makes them more … I don’t know how to say it, it is the controlling of the family can also result in negative mental health. And they often make the children and the other people in the family … more estranged and isolated … ‘ (WoRB 7)

‘There are still people who have to stay home, and they can’t do anything without the permission of the parents or husbands and things like that so…yeah, we do have people like that in our community.. who are still experiencing those kind of….the feeling of helplessness and loneliness and they can’t really do anything.. And also, the other thing is they are…. It is gender roles thing I guess; they also give care to sons and daughters…’ (WoRB 8)

The impact of originating from a country of origin that can be identified as patriarchal in nature was also identified as further disadvantaging WoRB in regard to engaging in an educational context, which in turn increased isolation and loneliness during resettlement in a regional location as it impacted their language acquisition skills.

‘English is very hard, many (WoRB) have never gone to school. They have always been a wife at home.’ (WoRB 5)

‘Language, that’s the main things, and the gender roles… and the feelings of isolation also comes from the language thing… like they can’t really express what they want to say, and in think that can make them feel more insecure maybe… so they often stay home and then make no contacts with other people. (WoRB 7)

‘Their English language acquisition is going to be so slow - because they’ve had extremely limited access to education or English before. (Service Provider 13)

‘Their traditional culture usually excludes them from education, possibly, and some of them have never been educated in their own country and in their own language, they can’t write or can’t read, so … to then come to a new country, having all the issues that they’ve brought with them, then to be learning a new language in our education system and our teaching ways, it’s totally foreign, completely foreign (Service Provider 3)

### Trauma stemming from the resettlement process

WoRB highlighted the significant negative impact that the resettlement process and initial resettlement period had on their mental health, with several WoRB identifying this period as traumatic in nature.

‘So when I arrived I wasn’t eligible for, like, the settlement services that most others did, so ... and I fell through the system. And it’s really hard. I guess, when you think about mental health, for me anyway, so like, there’s that trauma of migrating, itself, and even if you’re, like, a more economically well-off migrant, there’s still the trauma of leaving your home behind and moving to a new country (WoRB 1)

‘Trying to come to a different country like Australia was very hard, it is very hard. Your living in someone’s country, and they class you as a refugee, and they don’t allow you access to good education, and there is a bit of racism and people treat you a bit different’. (WoRB 3).

‘That migration is one aspect of that trauma, and like, the impacts on mental health, and then within that space of change, you also experience a lot of change within your family unit and your family structure. So, it’s like a double-edged sword, almost, when it comes to seeking help or getting help, because you kind of tend to ... well, I feel like women generally tend to kind of prioritise family within that settlement or moving process, so I feel like there’d be a lot of impact, because there’s changing gender roles ... I imagine it would be depressing, anxiety inducing, and all kinds of things. (WoRB 4)

### Inability to access fundamental needs for everyday life

WoRB emphasised the negative impact, and significant stress, that a lack of access to basic fundamental needs during resettlement caused, in particular housing and financial strain.

‘I was in a [name removed] until they look for your house, but … the information that we get is you don’t stay more than 1-2 months in guest house, in the orientation they tell us that and when we come, I am ready to go after two months … and I call and I ask, now we are at two months, where is our home?... they say ‘not yet, not yet’… not yet, not yet… then it has been one year... A long time… for me it was stressful. (WoRB 2)

“Living in temporary accommodation for 12 months at the beginning was difficult. A house is more than something over your head, it is a place to call your own after having so much movement and uncertainty for so long’ (WoRB 4).

‘Centrelink does pay, but I have to decided what I am going to use to pay for this and this, so you don’t have money to spend. You don’t know how much money the electricity is, and this sort of stuff, so it causes a lot of stress and worry, because you don’t know how you’re going to pay for it.’ (WoRB 3).

The impact that a lack of access to and availability of basic human needs, such as stable shelter, was also highlighted by service providers supporting WoRB resettled in Tasmania. Furthermore, it was reported as one of the biggest factors impacting on WoRB mental health, and a core reason for accessing support via a service. Although difficulties in accessing basic human needs, such as housing, was reported as a prominent stressor for WoRB, service providers reported that they were often unable to meet the needs of women accessing their service

‘A lot of the women who came here on the Women At Risk visa, single mums with five or six children, I mean, trying to get a rental here, is nearly impossible. So, the stress of that … I mean, I think every day, I must be talking to someone distressed; housing is probably one of the biggest stresses for a lot of people at the moment’ (Service Provider 5)

‘It's housing, it's shelter, it's food. Um, it's understanding what this letter means, so that can be very, very challenging but it does demonstrate that the level of need is very high and not being necessarily matched (Service Provider 1)

‘Those necessities, those survival things, having access to food, having shelter, um, having a steady stream of income, um, community support, friends, family, really good’ (Service Provider 6)

### Family separation and ongoing concerns for family and friends in their country of origin

A significant concern, and ongoing mental health stressor, identified by WoRB stemmed from family separation, which resulted from situations where family members were left behind in their country of origin after the WoRB resettled in Australia. This ongoing concern and distress for family and friends in country of origin was magnified due to barriers in regular communication with those who remained in the country of origin due to lack of access to telecommunication services and being unable to financially support them due to the fiscal strain experienced by WoRB.

‘We worry about family. My dad in [country of origin]..and I can sometimes speak with him, but not often, because they do not have internet or good mobile… And my mum is far away for us…. and sometimes talking with them is difficult, because the day and night is different. But my dad, I worry about my dad, because I don’t have enough money to help him’. (WoRB 5)

‘Some people often had to leave their family back in the country or often when they have families and relatives in different countries around the world… that also makes them more isolated. And they often think and worry about other people living in other countries, which can make them very sad and not want to talk to anyone.. it’s feeling the loss of family (WoRB 7)

Service Providers also identified the profound negative impact that separation from family had on mental health in WoRB. In particular, ongoing conflict in the WoRB country of origin was identified as significantly impacting the mental health of WoRB during resettlement

‘Throughout their whole experience is continued grief and loss over family and loved ones still back in their country of origin and, you know, we need to be proactive when we hear of, um, bombing in Syria, it's like contacting our clients who are in that space because the impacts that that has in the everyday is profound for them (Service Provider 1).

‘I’ve worked with a lot of women who’ve had to leave children behind. To come here with some children, leave some behind. And they, they just get … it must be absolutely heartbreaking for them. But in some cases, they’re actually becoming so upset and depressed about it, that they’re not actually looking after their children they do have with them, and, and able to settle well in Australia because they’re permanently grieving for the children that they’ve lost behind, left behind. (Service Provider 5).

### Chronic uncertainty

Service Providers highlighted the substantial negative impact that insecurity of tenure, due to being on a temporary protection visa, were having on the mental health of WoRB resettled in Tasmania. The uncertainly surrounding their future in Australia was identified as placing WoRB in a constant state of ‘limbo’, and contributing to the aforementioned trauma associated with resettlement

‘The detrimental effects of years of being in limbo – You know, we’ve seen a lot of women come through here with depression and anxiety because there’s no – there’s no certainty about anything. They leave their country of origins due to quite horrific traumas but sometimes it’s the trauma that we put on someone in the limbo and not knowing. Like this one woman– we heard today that she’s gone in to have her children have their medicals, which is a really positive step because they won’t let you do that if you’re not getting a visa , but it’s been probably been four years of limbo… that is an especially long time to have no security’. (Service Provider 11)

‘Coming here and feeling, you know, different levels of security, I suppose, because for `the-the women that I’ve dealt with a lot of the time, they don’t know what their future’s going to be, it’s-it’s really just, you know, the destructive, um, difficult process for people’ (Service Prover 5)

The process of obtaining a visa, and the associated uncertainty and fear was also highlighted as impacting upon WoRB seeking and accessing mental health support, due to fear that it would reduce the likelihood that they would receive a visa

But people generally are frightened of admitting to any mental health issues because they think that that can affect their, the acceptance rate of their visa application (Service Provider 6).

## Discussion

This study explored how mental health is conceptualised by WoRB and highlighted key factors impacting on the mental health of WoRB resettled in a regional location in Australia. In particular, the current study found that WoRB conceptualised mental health as a pathogenic entity, associated with being ‘*crazy*’ or *‘something in your mind’,* often stemming from too much stress. The current study also identified that the negative connotations which WoRB associated with mental health had significant flow on impacts in regard to accessing mental health services for support during resettlement, in particular, being stigmatised for seeking help with a mental health disorder. This finding is consistent with research in both refugee and non-refugee populations, in which higher levels of mental health stigma has been associated with reduced help seeking behaviour [30, 31]. Further, in studies which have focused on refugee populations, it has been identified that stigma is a prominent barrier to help seeking due to fears surrounding the consequences for the self (‘*being taken to a crazy place’*), and family unit (due to seeking support being identified as shameful for the family unit) [32]. Uniquely, this study highlights the significant impact that stigma associated with mental health can have on seeking mental health support in rural and regional locations in Australia. This is due to a limited number of mental health services available to individuals of refugee background (in the current study, only one per major regional area), impacting anonymity, and increasing the likelihood that members of the same family, and cultural group may be accessing the service. This was identified as stopping an individual from seeking help entirely despite an identified need. This suggests that the current structure of delivering specialist mental health services in rural and regional locations in Australia to refugee populations is inadequate. The centralisation of mental health support to one service may reduce to likelihood of WoRB accessing support, due to fear of seeing someone from their family or community. This can be seen as a unique issue in rural and regional locations, as major metropolitan resettlement locations will often have several options in regard to mental health support via government and non-government agencies. This can be identified as a particular pressing concern with the Australian Government’s announcement to resettle 50% of new humanitarian arrivals to a regional location by 2022 [33].

The current study also highlighted the role that religion can have in contributing to stigma surrounding mental health, and subsequent help seeking. WoRB in the current study highlighted that mental illness can be associated with a lack of faith, or not participating enough in religious worship, such as praying. Based on this finding, it can be suggested that religion may act as a double-edged sword in the health and wellbeing of refugee populations during resettlement, when taken in conjunction with previous research identifying religious beliefs as not only being associated as a cause of mental illness [34], but also as being a vital factor associated with people’s wellbeing and resilience [35].

When exploring factors which influenced the mental health of WoRB resettled in regional Australia, WoRB and service providers supporting WoRB, identified a number of a factors. Interestingly, it is important to highlight that all factors identified in the current study occur in the post-migration phase of resettlement (despite the research question asking about factors influencing mental health in general, not restricted to a particular phase of migration). This suggests that post-migration factors have a significant and salient influence on WoRB mental health. The impact of post migration stressors on mental health is gaining greater attention within research focusing on refugee populations, with theoretical frameworks, such as the Triple Trauma Paradigm, highlighting not only the potential traumatic stressors which refugee populations experience in their country of origin and during migration, but also the traumatic stressors experienced during resettlement [36]. Overall, this highlights the importance of gaining a deeper understanding of these potentially traumatic stressors during resettlement, and developing an understanding around how these stressors experienced in the post-migration period can influence psychopathology, and help seeking behaviour in this highly vulnerable population [37]. It is also important to highlight that although a majority of the post migration stressors identified within the current are not unique to regional resettlement locations, regional resettlement locations may place WoRB at greater risk of experiencing them, as discussed below in further detail.

Loneliness was emphasised by WoRB as particularly impacting their mental health. This is consistent with several studies focusing of the mental health challenges associated with resettlement in rural and regional locations of Australia [17, 19], with loneliness being associated with a lack of social support, which places WoRB at increased risk of mental health disorders, due to removing a valuable adaptive and coping resource [35]. However, the current study offers a unique insight into how gender-based norms and patriarchal values from country of origin may particularly contributed to loneliness and isolation in WoRB during resettlement. This is due to women needing to seek permission to engage in activities outside the home, and often taking on the primary caregiving roles, reducing their opportunities to develop social connections, and slowing adaptation due to limited exposure to the dominant culture within the country of resettlement. Furthermore, WoRB in the current study highlighted how the limited opportunities to engage in education, including basic numeracy and literacy, in their country of origin significantly impacted their capacity to engage in educational contexts during resettlement, impacting their language acquisition skills and communication styles. Such gender-based challenges associated with engaging in education during resettlement need to be taken into consideration when considering resettlement supports for WoRB, particularly in regional and rural areas, as access to adequate language classes in regional locations has been identified as a key resettlement challenge [18]. Failure to take these gendered vulnerabilities into consideration within policy and programs aimed at supporting WoRB during resettlement runs the risk of perpetuating gender specific vulnerabilities in the country of resettlement, which are often entrenched in the patriarchal social structure of the WoRB country of origin [22].

A pressing concern in the current study, which was highlighted by not only WoRB, but also the service providers providing daily support to WoRB during resettlement was the lack of access to basic necessitates, such as housing and food. Lack of access to important basic necessitates can not only have devastating immediate and long-term impact (such as homelessness and malnutrition), but can also have significant interpersonal impacts, such as social exclusion, low social status and discrimination [38]. Research has shown that access to basic necessitates is more difficult in regional and rural location of Australia for the general population, with individuals living in regional and rural Australia experiencing higher levels of poverty, in comparison to individuals who live in metropolitan areas [39]. This suggests that regional and rural areas of Australia are already stretched, and significant changes to funding and service provision is needed prior to the increased allocation to newly settled humanitarian entrants to regional locations of Australia.

WoRB in the current study also highlighted the impact that ongoing separation from family had on their mental health during resettlement. The impact of family separation during resettlement has been identified in previous research as the most salient and significant source of ongoing stress and sadness during resettlement [40, 41]. Further, WoRB in the current study highlighted that they felt a pressure to financially support family members back home, despite having difficulty financially supporting their own households. The impact that the ongoing separation from family has on WoRB, and individuals of refugee background in general, warrants particular attention in rural and regional resettlement locations due to the Australian Government being more likely to resettle individuals of refugee background without family connections in Australia in these locations [21, 42, 43].

In addition to the impact of family separation on the mental health of WoRB resettled in a regional Australia, the current study also provides qualitative evidence surrounding the impact of ongoing uncertainty in regard to visa insecurity on the mental health of WoRB. This is consistent with emerging evidence that ongoing visa insecurity is an important predictive factor for individuals of refugee background showing symptoms of Complex Post Traumatic Stress Disorder and Adjustment Disorder during resettlement, stemming from a sense of loss of control and fear of being repatriated [44], and suggests that ongoing visa insecurity could be classified as a form of trauma within itself.

The findings of the current study have a number of implications, on both the service and policy level. The current study shows that WoRB experience additional challenges during resettlement, which place them at higher risk for experiencing ongoing stress. Despite this, this study suggests that some regional locations may not have adequate resources to support this vulnerable population. This is despite the Australian governments push to increase the number of individuals of refugee background resettling in regional Australia substantially over the next 2 years [33]. Currently, mental health services in regional Australia are pushed to the extremes, and do not have the resources, and limited knowledge on how to provide culturally sensitive care [45]. It is recommended that additional funding and training for service is needed to ensure that adequate, culturally sensitive mental health care and support can be provided in regional resettlement locations.

Based on the findings of the current study, it is also recommended that policy makers place greater emphasis on addressing the social determinates of health, which are significantly impacting on the mental health of WoRB during resettlement. By alleviating stressors associated with social determinates of health, such as housing, access to adequate food and providing more flexible education options, the stressors which WoRB identified as adversely impacting of their mental health may alleviate, which in turn may result in overall better wellbeing, and a higher quality of life in regional resettlement locations.

The current study has several limitations. Firstly, to participate in the current study, all WoRB were required to have an adequate level of conversational English, as all interviews were conducted in English. This likely limited to opportunity for some WoRB to participate in the interviews, particularly those more recently resettled who are less likely to have developed strong English language skills. As such, it is not clear whether the experiences detailed by participants are representative of the entire resettlement process, or those most salient at later stages of the resettlement process. Longitudinal designs would assist in determining whether the process of resettlement change as a function of time and phase of adaptation. Secondly, the current study also used convenience sampling, which may detract from the representativeness of the sample, and therefore the overall generalisability of the findings. Finally, the current study did not collect participant demographic information. This was a deliberate decision to reduce the likelihood of participants being identified, and beaching confidentiality. This is particularly pertinent in the current study due to it being conducted in a regional location and some ethnic groups only having two-three families living in the regional location. Therefore, identifying that a WoRB from a particular ethnic group participated in the study would have significantly impacted on their anonymity. Despite this, collecting and reporting demographic information would have provided important information pertaining to the generalisability of the current study population to future research, thus it must be acknowledged as a limitation’.

## Conclusion

This study is the first to specifically focus on the mental health of WoRB resettled in a regional location of Australia. The findings build upon existing research which indicates the adverse impacts post-migrations stressors can have on the mental health of individuals of refugee background. Furthermore, this study suggests that the current services and supports available to WoRB resettled in regional locations of Australia are inadequate, and under-resources.

## Data Availability

The datasets generated and/or analysed during the current study are not publicly available due to ethical restrictions imposed by the University of Tasmania and Charles Darwin University Research Ethic Boards as public availability would compromise participant confidentiality and privacy, but are available from the corresponding author on reasonable request. Due to ethical restrictions related to protecting the privacy imposed by the University of Tasmania and Charles Darwin University Research Ethic Boards, the full, qualitative dataset (transcripts and field notes) cannot be made public. Public availability would compromise participant confidentiality and privacy.

## References

[1] Freedman J (2016). Sexual and gender-based violence against refugee women: a hidden aspect of the refugee “crisis”. Reprod Health Matters.

[2] Shishehgar S, Gholizadeh L, DiGiacomo M, Green A, Davidson PM (2017). Health and socio-cultural experiences of refugee women: an integrative review. J Immigr Minor Health.

[3] Mangrio E, Zdravkovic S, Carlson E (2019). Refugee women’s experience of the resettlement process: a qualitative study. BMC Womens Health.

[4] Cooper S, Enticott JC, Shawyer F, Meadows G (2019). Determinants of mental illness among humanitarian migrants: longitudinal analysis of findings from the first three waves of a large cohort study. Front Psychiatry.

[5] Schweitzer R, Melville F, Steel Z, Lacherez P (2006). Trauma, post-migration living difficulties, and social support as predictors of psychological adjustment in resettled Sudanese refugees. Aust N Z J Psychiatry.

[6] Blackmore R, Boyle JA, Fazel M, Ranasinha S, Gray KM, Fitzgerald G, Misso M, Gibson-Helm M (2020). The prevalence of mental illness in refugees and asylum seekers: a systematic review and meta-analysis. PLoS Med.

[7] Schweitzer RD, Vromans L, Brough M, Asic-Kobe M, Correa-Velez I, Murray K, Lenette C (2018). Recently resettled refugee women-at-risk in Australia evidence high levels of psychiatric symptoms: individual, trauma and post-migration factors predict outcomes. BMC Med.

[8] Jarallah Y, Baxter J (2019). Gender disparities and psychological distress among humanitarian migrants in Australia: a moderating role of migration pathway?. Confl Heal.

[9] Due C, Burford-Rice R, Augoustinos M, Denson L. Help-seeking for mental health services in Australia among Afghan women with refugee backgrounds. Eur J Pub Health. 2018: OXFORD UNIV PRESS GREAT CLARENDON ST, OXFORD OX2 6DP, ENGLAND.;28(suppl_1). 10.1093/eurpub/cky047.050.

[10] Kronick R (2018). Mental health of refugees and asylum seekers: assessment and intervention. Can J Psychiatr.

[11] Baird MB, Cates R, Bott MJ, Buller C (2020). Assessing the mental health of refugees using the refugee health Screener-15. West J Nurs Res.

[12] Vromans L, Schweitzer RD, Brough M, Correa-Velez I, Murray K, Lenette C (2018). Psychometric properties of the multidimensional loss scale with refugee women-at-risk recently arrived in Australia. J Immigr Minor Health.

[13] Council UNHR (2013). The integration of resettled refugees: essentials for establishing a resettlement Programme and fundamentals for sustainable resettlement Programmes.

[14] Bartolomei L, Eckert R, Pittaway E (2014). “What happens there... follows us here”: Resettled but Still at Risk: Refugee Women and Girls in Australia. Refuge.

[15] Australian Government (2020). 2018–19 humanitarian program outcomes: Department of Home Affairs.

[16] O’Neil A (2019). House of assembly select committee on housing affordability.

[17] Hamrah MS, Hoang H, Mond J, Pahlavanzade B, Charkazi A, Auckland S. The prevalence and correlates of symptoms of post-traumatic stress disorder (PTSD) among resettled afghan refugees in a regional area of Australia. J Ment Health. 2020:1–7. 10.1080/09638237.2020.1739247. 10.1080/09638237.2020.1739247 32223476

[18] Smith L, Hoang H, Reynish T, McLeod K, Hannah C, Auckland S, Slewa-Younan S, Mond J (2020). Factors shaping the lived experience of resettlement for former refugees in regional Australia. Int J Environ Res Public Health.

[19] Hamrah MS, Hoang H, Mond J, Pahlavanzade B, Charkazi A, Auckland S. Occurrence and correlates of depressive symptoms among the resettled afghan refugees in a regional area of Australia. Early Intervention Psychiatry. 2020:1–7. 10.1080/09638237.2020.1739247. 10.1111/eip.12957 32243096

[20] Services AME (2011). Regional settlement report: an investigation of four regional settlement locations in Victoria.

[21] Vasey K, Manderson L (2012). Regionalizing immigration, health and inequality: Iraqi refugees in Australia. Adm Sci.

[22] Sullivan C, Vaughan C, Wright J (2020). Migrant and refugee women’s mental health in Australia: a literature review.

[23] Australian Bureau of Statistics (2019). 2016 Census QuickStats.

[24] Australian Bureau of Statistics (2016). Tasmania Census Statistics.

[25] Clark-Kazak C (2017). Ethical considerations: research with people in situations of forced migration. Refuge.

[26] Block K, Warr D, Gibbs L, Riggs E (2013). Addressing ethical and methodological challenges in research with refugee-background young people: reflections from the field. J Refug Stud.

[27] Sabouni F (2019). Exploring the psychosocial needs of Syrian refugees in the UK: accounts of community service providers: University of Manchester.

[28] Braun V, Clarke V (2006). Using thematic analysis in psychology. Qual Res Psychol.

[29] Tong A, Sainsbury P, Craig J (2007). Consolidated criteria for reporting qualitative research (COREQ): a 32-item checklist for interviews and focus groups. Int J Qual Health Care.

[30] Satinsky E, Fuhr DC, Woodward A, Sondorp E, Roberts B (2019). Mental health care utilisation and access among refugees and asylum seekers in Europe: a systematic review. Health Policy.

[31] Clement S, Schauman O, Graham T, Maggioni F, Evans-Lacko S, Bezborodovs N, Morgan C, Rüsch N, Brown JSL, Thornicroft G (2015). What is the impact of mental health-related stigma on help-seeking? A systematic review of quantitative and qualitative studies. Psychol Med.

[32] Byrow Y, Pajak R, Specker P, Nickerson A (2020). Perceptions of mental health and perceived barriers to mental health help-seeking amongst refugees: a systematic review. Clin Psychol Rev.

[33] Australian Government (2019). Investing in refugees, investing in Australia: the findings of a review into integration, employment and settlement outcomes for refugees and humanitarian entrants in Australia.

[34] Bettmann JE, Penney D, Clarkson Freeman P, Lecy N (2015). Somali refugees’ perceptions of mental illness. Soc Work Health Care.

[35] Hawkes C, Norris K, Joyce J, Paton D (2020). Resilience factors in women of refugee background: a qualitative systematic review. Community Psychol Glob Perspect.

[36] Fernando Chang-Muy J, Congress EP (2015). Social work with immigrants and refugees: legal issues, clinical skills, and advocacy: springer publishing company.

[37] Li SS, Liddell BJ, Nickerson A (2016). The relationship between post-migration stress and psychological disorders in refugees and asylum seekers. Curr Psychiatry Rep.

[38] Hynie M (2018). The social determinants of refugee mental health in the post-migration context: a critical review. Can J Psychiatr.

[39] Alliance NRH (2017). Poverty in Rural and Remote Australia.

[40] Miller A, Hess JM, Bybee D, Goodkind JR (2018). Understanding the mental health consequences of family separation for refugees: implications for policy and practice. Am J Orthop.

[41] Savic M, Chur-Hansen A, Mahmood MA, Moore V (2013). Separation from family and its impact on the mental health of Sudanese refugees in Australia: a qualitative study. Aust N Z J Public Health.

[42] Correa-Velez I, Barnett AG, Gifford SM, Sackey D (2011). Health status and use of health services among recently arrived men with refugee backgrounds: a comparative analysis of urban and regional settlement in south-East Queensland. Aust J Prim Health.

[43] McDonald-Wilmsen B, Gifford SM, Webster K, Wiseman J, Casey S (2009). Resettling refugees in rural and regional Australia: learning from recent policy and program initiatives. Aust J Public Adm.

[44] Liddell BJ, Nickerson A, Felmingham KL, Malhi GS, Cheung J, Den M (2019). Complex posttraumatic stress disorder symptom profiles in traumatized refugees. J Trauma Stress.

[45] Hawkes C, Norris K, Joyce J, Paton D (2021). Resettlement stressors for women of refugee background resettled in regional Australia. Int J Environ Res Public Health.

